# Systematic Benchmarking of Spectral Demodulation Methods for Ball Resonator and Hybrid FPI–Ball Resonator Sensors for Multiparameter Physiological Monitoring

**DOI:** 10.3390/bios16050278

**Published:** 2026-05-11

**Authors:** Natsnet Bereket Tecle, M. Fátima Domingues

**Affiliations:** 1 Department of Biomedical Engineering and Biotechnology, Khalifa University of Science & Technology, Abu Dhabi 127788, United Arab Emirates; 100053693@ku.ac.ae; 2 Healthcare Engineering and Innovation Group, Khalifa University of Science & Technology, Abu Dhabi 127788, United Arab Emirates; 3 Instituto de Telecomunicações, University of Aveiro, 3810-193 Aveiro, Portugal

**Keywords:** ball resonator sensor, Fabry–Pérot interferometer (FPI), hybrid optical fiber sensor (OFS), Karhunen–Loève transform (KLT), multiparameter sensing, spectral demodulation

## Abstract

Ball resonator optical fiber sensors (OFSs) can offer multiparameter sensing capability, but their non-periodic and low-finesse reflection spectra make conventional spectral demodulation unreliable. This work proposes two sensor configurations: (i) a ball resonator and (ii) a hybrid sensor integrating a Fabry–Pérot interferometer (FPI) with a ball resonator, and compares their performance for multiparameter physiological monitoring using the Karhunen–Loève transform (KLT). The sensors were evaluated for glucose concentration (0–3 mg/mL), temperature (20–55 °C), and pH (3–9) monitoring. The ball resonator sensor, paired with KLT, achieved high linearity across all measurands (R^2^ = 0.989, 0.919, and 0.838 in response to glucose, temperature, and pH, respectively). The hybrid sensor exhibited a higher glucose sensitivity (6.15 a.u./(mg/mL)) compared to the ball resonator (3.77 a.u./(mg/mL)), resulting in limits of detection (LODs) of 2.53 mM and 4.19 mM, respectively. In contrast, the ball resonator sensor demonstrated better sensitivity for temperature and pH sensing. Furthermore, we present a comprehensive benchmarking framework of seven spectral demodulation methods for OFSs. The results demonstrated that KLT consistently provides robust demodulation performance and highlighted the potential of KLT for multiparameter physiological sensing applications.

## 1. Introduction

Many diseases, including diabetes, sepsis, cancer, cardiovascular dysfunction, and wound infection, require accurate and real-time monitoring for early diagnosis and continuous management. Modern healthcare increasingly relies on simultaneous monitoring of key physiological parameters, such as glucose, temperature, and pH. Abnormal variations in these parameters can indicate hyperglycemia in diabetes, acidosis and fever in sepsis, acidic tumor microenvironments in cancer, altered thermal regulation in cardiovascular disease, and infection during wound healing [1]. However, existing electronic sensors often suffer from electromagnetic interference, limited miniaturization, poor compatibility with harsh environments, and restricted multiplexing capability [2]. Optical fiber sensors (OFSs) overcome these limitations by offering resistance to electromagnetic interference, compactness, high sensitivity, biocompatibility, and strong multiplexing capacity [2,3,4]. Among OFSs, ball resonator (microsphere) sensors have been receiving increasing interest, owing to their simple fabrication, mechanical stability, and potential for integration into compact lab-on-a-chip and point-of-care diagnostic systems [5,6,7,8].

**Figure 1:**
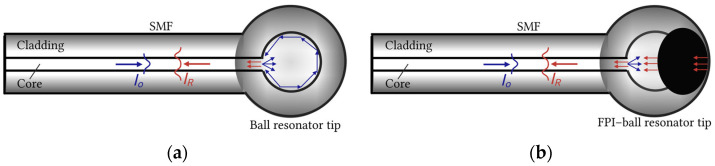
2-dimensional (2D) schematics illustrating light propagation in: (**a**) the ball resonator sensor, where light launched from the SMF core enters the spherical tip, undergoes multiple total internal reflections at the spherical boundary and forms a circulating optical field; and (**b**) the hybrid FPI–ball resonator sensor, where the incident light is reflected at the FPI and microsphere interfaces.

In conventional whispering gallery mode (WGM) resonators, light is typically coupled into the cavity using tapered fibers or prism couplers. Alternatively, ball resonators can operate as in-fiber integrated resonators, where light traveling along the core of a single-mode fiber (SMF) couples into the spherical tip [7,9], as shown in Figure 1a. When light reaches the curved sphere boundary at sufficiently shallow angles, it circulates as a WGM confined by total internal reflection (TIR) around the sphere’s equator [6]. However, the TIR is not perfect; a small portion of the light leaks out as an evanescent field due to curvature-induced losses. This evanescent field extends into the surrounding environment, enabling interaction with the external medium [10].

Ball resonator sensors exploit light–matter interactions to detect biochemical analytes, such as glucose and pH, where the molecular interactions and varying ionization states modulate the effective RI and induce significant spectral shifts [11,12]. Ball resonators have been demonstrated as refractive index (RI) sensors [5,13], biosensors [14], and further functionalized for immunosensing applications [15,16,17]. These studies established the sensor design and spectral interrogation in reflection-mode.

Ball resonators are also inherently sensitive to temperature through thermo-optic (change in silica’s RI) and thermal expansion effects (change in the radius of the microsphere), both of which induce significant spectral shifts. Several studies have demonstrated temperature-dependent spectral shifts in the transmission spectra of silica-fused microspheres [18,19,20]. In reflection-mode, ball resonator sensors can be applied for one-end biomedical temperature monitoring in environments where electronic sensors are unsuitable, such as magnetic resonance imaging systems and microwave ablation setups during chemotherapy.

The response of an OFS is often quantified by tracking a specific spectral feature, such as amplitude, frequency, phase, intensity, or dip/peak wavelength [3]. However, in reflection-mode, fiber-tip ball resonator sensors produce low-finesse, quasi-random spectral fringes in the range of −60 to −40 dB [5,16,17]. This spectral behavior arises from the mode-field mismatch due to inefficient coupling of the SMF directly into the microsphere [21]. Inconsistencies in ellipticity, surface reflectivity, and variable length of the fiber extenders contribute to the bending losses and randomness of the interference pattern [17,22]. As a result, a single spectral feature cannot fully capture the response of a ball resonator sensor. Nevertheless, reflection-mode interrogation enables one-end sensing, which is highly advantageous for in vivo or microfluidic applications [3,23,24].

Conventional demodulation methods include centroid or peak tracking, fixed-wavelength intensity change, spectral correlation, and fast Fourier transform (FFT)-based phase analysis. Previous studies have investigated the importance of applying advanced demodulation methods to enhance the performance of OFSs. Tosi et al. introduced the undersampled Karhunen–Loève transform (KLT) to reliably track changes in RI using ball resonator sensors [5]. KLT is a mathematical algorithm, similar to principal component analysis (PCA), that projects a signal onto an orthogonal basis of eigenfunctions derived from the autocorrelation matrix and extracts the dominant spectral components that contain most of the energy in the spectrum [5,25]. It has also been validated for fiber Bragg grating (FBG) and FPI sensors, showing significant improvements in accuracy and robustness to noise [26]. Unlike the conventional demodulation methods, PCA and KLT operate on the entire spectrum without the manual identification of a single spectral feature [26,27]. Because KLT captures the optimal basis of the entire spectrum from the autocorrelation function of the underlying random process, it is well suited for ball resonators, whose spectra are low-finesse and non-periodic [28,29].

To the best of our knowledge, Domingues et al. is the only study that has demonstrated a hybrid OFS integrating a fiber-tip Fabry–Pérot interferometer (FPI) with a ball resonator [30]. The FPI–ball resonator sensor was applied to monitor RI changes induced by varying concentrations of protein aggregates. The sensor response relied on changes in the amplitude of the reflected spectrum, achieving a performance comparable to fluorescence-based aggregation assays and highlighting the potential of the sensor for physiological and biomedical monitoring. The hybrid configuration forms a multi-cavity interferometric structure and exhibits a high spectral contrast [30]. As demonstrated in Figure 1b, the incident light undergoes multiple reflections at the SMF–resonator interface and the interfaces defining the FPI microcavity. Consequently, the reflected spectrum results from the interference among the multiple reflections.

This study aims to evaluate the reliability of full-spectrum analysis for spectral demodulation in OFSs, compared to conventional feature-tracking methods. To achieve this, we compare the performance of a ball resonator and a hybrid FPI–ball resonator sensor for monitoring glucose, temperature, and pH using KLT. The hybrid sensor is employed to enhance the spectral contrast and improve the observability of measurand-induced spectral variations. To the best of our knowledge, the hybrid FPI–ball resonator sensor has only been reported once in the literature and has not been applied to multiparameter physiological monitoring. This work establishes a comprehensive benchmarking framework of seven demodulation techniques, namely, centroid wavelength shift, intensity change, Pearson correlation, phase correlation, PCA, root mean square (RMS), and KLT. We present one of the most comprehensive benchmarking analyses of demodulation methods in OFSs. Furthermore, RMS is introduced as a spectral demodulation method for OFSs, having previously been used for real-time arc welding monitoring [31]. The results demonstrate the effectiveness of KLT-based full-spectrum analysis for multiparameter sensing.

## 2. Materials and Methods

### 2.1. Fabrication of Sensors

**Figure 2:**
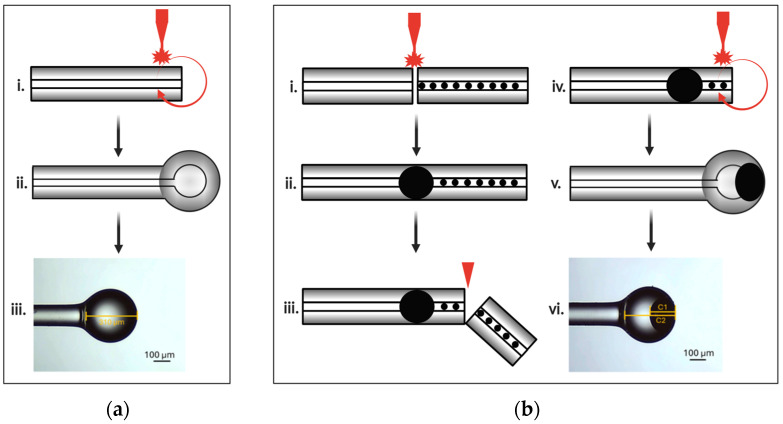
Fabrication process of (**a**) ball resonator: (**i**) alignment, rotation, and arc discharge applied for localized melting of the SMF tip; (**ii**) resulting ball resonator; (**iii**) microscope image of the fabricated ball resonator; and (**b**) FPI–ball resonator: (**i**) alignment and fusion splicing of the void-containing damaged fiber to a standard SMF; (**ii**) formation of the FPI microcavity at the splice point; (**iii**) cleaving to remove the damaged fiber segment beyond the cavity; (**iv**) alignment, rotation, and arc discharge at the fiber tip to form the ball resonator; (**v**) resulting hybrid FPI–ball resonator structure; (**vi**) microscope image of the fabricated hybrid sensor showing the FPI cavity (C1) and the microsphere (C2). Black arrows indicate fabrication progress, red curved arrows indicate fiber rotation, red electrode symbols indicate arc discharge, and the red wedge indicates fiber cleaving.

Unlike the fabrication method that relies on fusing two fibers and pulling them apart [32], ball resonators can be fabricated directly on the tip of a single SMF. The Fibermart Corning SMF-28e+ (Fibermart Company Inc., Hong Kong, China) was cleaved and mounted on a Fujikura FSM-100P+ fusion splicer (Fujikura Ltd., Tokyo, Japan) operating in fiber shaping mode. This approach eliminates the splicing step and forms the spherical tip directly. The controlled arc discharge melts the silica end, and the resulting surface tension forms a sphere. During arc discharge, the fiber is rotated to ensure uniform heating and re-shaping of the molten silica. The arc power and time were set to 311 bit and 2000 ms, respectively. Higher arc power and longer arc duration increase the amount of silica melted at the fiber tip, forming larger and more spherical microspheres with smoother surfaces. However, excessive heating may degrade surface quality through over-melting. The fusion splicer’s preset NS F125 B300 was selected to produce the microsphere. The resulting monolithic silica microsphere with a diameter of 310 µm is shown in Figure 2a(iii). The fusion splicer parameters and their corresponding values are provided in Table S1.

The hybrid FPI–ball resonator sensor was produced using a two-step process. First, the FPI was formed following the procedure previously described in [30,33]. This step involves splicing a fiber containing small, periodic voids into a standard Fibermart Corning SMF-28e+ using Jilong KL-530 fusion-splicer (Nanjing Jilong Optical Communication Co., Ltd., Nanjing, China). The void-containing fiber was obtained from an SMF (Fibermart Corning SMF-28e+) previously damaged by the catastrophic fuse effect. During fusion-splicing, the silica around some of the voids softens and collapses, creating an elliptical or teardrop-shaped air-filled microcavity, as shown in Figure 2b(ii). The cavity length is determined by the size of the collapsed voids during fusion splicing. Prior to microsphere formation, the damaged fiber segment beyond the cavity was removed by cleaving the fiber approximately 0.3 mm from the cavity. In the second step, the ball resonator was formed at the cleaved tip of the same fiber following the steps described above for a ball resonator using Fujikura FSM-100P+ fusion splicer. The resulting structure is a dual-cavity interferometer consisting of the FPI cavity (C1) with an axial cavity length of 139 µm and a microsphere (C2) with a diameter of 310 µm. The fabricated hybrid sensor is shown in Figure 2b(vi).

### 2.2. Experimental Setup

The sensors were interrogated using a Luna Micron Optics HYPERION si155 interrogator (Luna Innovations Inc., Roanoke, VA, USA) with a wavelength range of 1500–1600 nm and a resolution of 0.01 nm. All chemicals were obtained from Sigma Aldrich (St. Louis, MO, USA).

For glucose sensing, aqueous D-(+)-glucose solutions were prepared at concentrations ranging from 0 to 3 mg/mL. This range spans fasting glucose levels and extends into hyperglycemic concentrations. It provides a broad RI variation for sensor evaluation. Because detection relies on the intrinsic increase in RI with glucose concentration, a KERN ORL 94BS digital refractometer (KERN & SOHN GmbH, Balingen, Germany) was used to measure the RI of each solution prior to testing. The prepared glucose solutions exhibited a consistent RI increase in the order of 2 × 10^−4^ RIU/(mg/mL), which is sufficient to generate significant spectral shifts (Figure S1).

For pH sensing, solutions spanning pH 3–9 were prepared by titrating distilled water with hydrochloric acid (HCl) and sodium hydroxide (NaOH). This range covers the pH values of most physiological fluids (typically pH 5–8), including the more extreme pH values reported in pathological and wound microenvironments. Prior to testing, the pH of each solution was validated using a calibrated Milwaukee MW102 pH meter (Milwaukee Instruments, Rocky Mount, NC, USA).

For both glucose and pH measurements, the spherical fiber-tips were fully submerged into each solution sequentially, and the reflection spectra were recorded over repeated measurements (*n* = 5). Therefore, each data point represents the mean value of the repeated measurements and error bars correspond to the standard deviation. To avoid cross-contamination, the sensors were rinsed with distilled water and dried between measurements. All experiments were performed at room temperature to minimize thermal drift. Statistical analysis was performed using linear regression. The statistical significance of the slope was assessed using a two-tailed *t*-test. A significance level of *p* < 0.05 was considered statistically significant throughout, and 95% confidence intervals (CI) were calculated for the fitted parameters.

**Figure 3:**
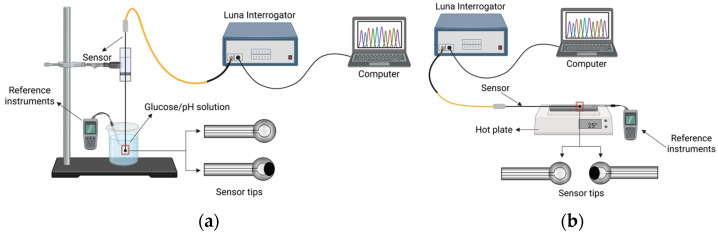
Schematic representation of the experimental setup and the signal interrogation system in reflection-mode using a HYPERION si-155 Luna interrogator connected to a computer. (**a**) For glucose and pH measurements, the sensor tips were immersed in prepared glucose and pH solutions while reference measurements (refractometer and pH meter) were acquired; and (**b**) for temperature characterization, the sensors were placed in direct contact with the hotplate surface while a digital thermometer was used as the reference.

Temperature characterization was performed by placing the sensors on a Cimarec+™ Thermo Scientific digital hotplate (Thermo Orion Inc., Chelmsford, MA, USA) and varying the temperature from 20 °C to 55 °C in 2 °C increments. The selected temperature range spans room-temperature conditions, physiological temperatures, and the elevated temperatures encountered during hyperthermia and thermal therapies such as microwave ablation. The measurements were repeated three times over the range of 20–55 °C. For each run, the sensor response at each temperature was referenced to the baseline value at 20 °C to compensate for potential signal drift between runs. Consequently, the reported values represent the mean change in temperature relative to the 20 °C baseline, and the error bars indicate the corresponding standard deviation across runs. To ensure accuracy, the hotplate temperature was verified using a digital thermometer. Thermal stability was maintained by enclosing the sensors within a thermal insulation box during all measurements. The complete experimental setup is shown in Figure 3.

### 2.3. Spectral Demodulation Methods

**Figure 4:**
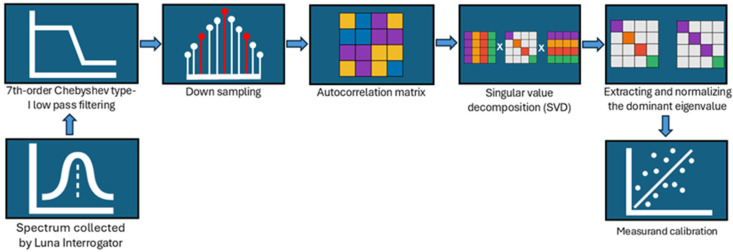
Spectral demodulation pipeline based on undersampled KLT. The reflection spectrum is first filtered using a 7th-order Chebyshev type-I low-pass filter and then downsampled. The autocorrelation matrix of the spectrum is constructed and decomposed using singular value decomposition (SVD). The dominant eigenvalue is extracted and normalized by the number of samples in the downsampled spectrum. The final output, *ω*, is used for measurand calibration to obtain the sensor response.

The acquired spectra were analyzed to evaluate the response of the sensors to the three measurands. KLT-based demodulation followed the approach discussed by Tosi et al. [5], and the overall pipeline is illustrated in Figure 4. The changes in the KLT output (*ω*) of each spectrum were monitored and correlated with variations in the measurands.

To benchmark the performance of KLT, six additional demodulation methods were implemented, each designed to capture a distinct aspect of the spectral response. As in the KLT analysis, the reflection spectra were first filtered using a 7th-order Chebyshev type-I low-pass filter. The spectra measured at 0 mg/mL (glucose), 20 °C (temperature), and pH 3 served as the baseline for comparison. The methods were:

- Centroid wavelength shift—tracks changes in the barycenter (intensity-weighted average wavelength) of the spectrum with variations in the measurands [34].
- Intensity change—measures the variations in intensity at a fixed wavelength [35].
- Pearson correlation—quantifies the degree of similarity between each spectrum and the corresponding baseline spectrum [36].
- Phase correlation—estimates the global wavelength shift (lag) of each spectrum through cross-correlation with the baseline [37].
- PCA—extracts the principal component of the spectrum and tracks its changes with variations in the measurands [27].
- RMS—provides a complementary view, evaluating the variation in the overall spectral power with changes in the measurands [31].

For each method, sensitivity (slope) and linearity (R^2^) were computed through linear regression. This comprehensive benchmarking framework enables a systematic assessment of the robustness of KLT relative to the other spectral demodulation methods. All spectral analysis was performed on a MacBook Air with 16 GB RAM in MATLAB R2024b (MathWorks Inc., Natick, MA, USA).

## 3. Results and Discussion

### 3.1. Reflection Spectra of the Sensors

In optical resonant cavities, the spectral response is often described by a Lorentzian resonance model. Lorentzian resonances are symmetric and arise in ideal WGM resonators where background reflections are negligible. However, in practice, non-resonant paths, such as Fresnel reflections and scattering from surface imperfections, introduce a background reflection [8]. The interaction between the discrete WGM resonant modes and the background reflection gives rise to constructive and destructive interference, distorting the Lorentzian profile into an asymmetric Fano resonance [38,39]. Fano resonances exhibit irregular dips and peaks, as well as flattened spectral features [38].

**Figure 5:**
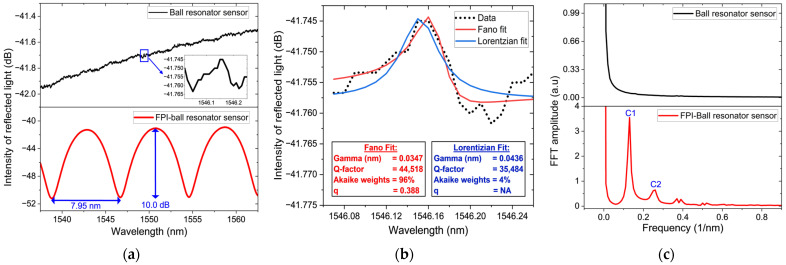
Spectral analysis of the ball resonator and hybrid FPI–ball resonator sensors: (**a**) collected reflection spectra; (**b**) Fano (red) and Lorentzian (blue) fit models of the resonance of the ball resonator sensor near 1546 nm; and (**c**) FFT spatial-frequency spectra.

The reflection spectra of the produced sensors are shown in Figure 5a. The two sensors exhibit distinct spectral patterns. The hybrid FPI–ball resonator sensor displays a well-defined interference pattern dominated by the FPI cavity, with high-contrast and periodic sinusoidal fringes. The measured free spectral range (FSR) and fringe visibility are 7.95 nm and 10.00 dB, respectively. In contrast, the ball resonator sensor shows a weak, low-contrast spectrum in the −43 to −41 dB range, consistent with low-contrast behavior reported in [5,13,16]. In in-fiber (integrated) resonators, the coupling mechanism is weaker than conventional coupling schemes. The low-contrast spectrum arises from weak coupling between the light-guiding mode in the SMF and the microsphere [39].

The interference between the resonant modes and background reflections produces asymmetric Fano-type spectral features [39]. The inset in Figure 5a highlights the Fano asymmetry in the reflection spectrum of the ball resonator sensor. The resonance near 1546.16 nm is fitted using both Lorentzian and Fano models, as shown in Figure 5b. The FWHM and Q-factor of the Fano model are approximately 0.0347 nm and 4.45 × 10^4^, respectively. The reported Q-factor is comparable to values reported for in-fiber WGM microsphere resonators, which typically range from 10^4^ to 10^5^ [39]. Model selection using Akaike weights favors the Fano model with a 96% probability of being the best model for representing the experimental data. This confirms that the resonance is asymmetric. The extracted asymmetry parameter (*q* = 0.39) also supports a strong asymmetric profile, since smaller values represent an asymmetric Fano spectrum [40].

Corresponding spatial frequency spectra obtained via FFT are shown in Figure 5c. The FFT analysis provides further insights into the frequency-domain characteristics of the two sensors. The ball resonator sensor exhibits a broad and distributed frequency response without any sharp frequency peaks. This reflects the low-visibility and quasi-random nature of its reflection spectrum. Although this behavior complicates direct spectral tracking, it provides a unique spectral fingerprint highly sensitive to environmental changes [5,39]; therefore, advanced spectral demodulation is required. In contrast, the hybrid FPI–ball resonator sensor shows strong and sharp spatial frequency components. A strong peak appears at approximately 0.13 1/nm, corresponding to the FPI cavity (C1), while a second peak at around 0.26 1/nm (C2) arises from the ball resonator cavity.

### 3.2. KLT Computational Efficiency

**Figure 6:**
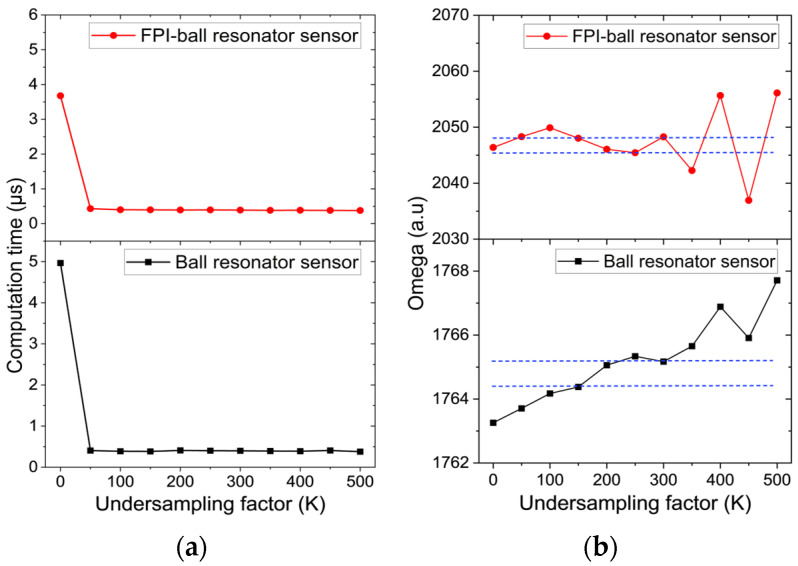
Computational efficiency of the undersampled KLT applied to the ball resonator and hybrid FPI–ball resonator sensors: (**a**) computation time as a function of the undersampling factor (*K*); and (**b**) effect of *K* on the dominant KLT eigenvalue (*ω*), where the blue horizontal dashed lines mark the 0.1% variation bounds of *ω*.

To evaluate the computational efficiency of KLT, the reflection spectra acquired at room temperature in air were analyzed. Figure 6a shows the computation time as a function of the undersampling factor (*K*). For both sensors, the computation time decreases rapidly to below 0.5 µs as *K* increases from 0 to 50. This behavior occurs as fewer spectral points are processed by the KLT algorithm as *K* increases. Beyond this point, the computation time remains essentially constant, indicating that further undersampling provides minimal additional computational benefit.

The computation times obtained lie in the microsecond range, substantially faster than those reported by Tosi et al. [5], where computation times fell below 10 ms at *K* = 200. This difference arises from the spectral resolution of the interrogation devices employed: Tosi et al. [5] used an optical backscatter reflectometer (OBR 4600) with a resolution of 0.02 pm [41], whereas the present study employs a HYPERION si155 interrogator with a lower resolution of 10 pm [42]. The lower spectral resolution results in significantly fewer sampled data points per spectrum, thereby yielding much shorter KLT computation times.

Undersampling reduces redundancy in the spectrum and increases computational speed. However, very high undersampling factors can remove key spectral information and degrade performance. To identify an optimal balance between computational speed and accuracy, the evolution of *ω* was analyzed as a function of *K* (Figure 6b). Following the criterion in [5], keeping *ω* within a 0.1% deviation ensures that KLT effectively extracts a stable spectral feature. Based on this threshold, 150 < *K* < 300 is identified as the optimal undersampling boundary. Therefore, *K* = 200 is applied for all subsequent analyses.

### 3.3. Glucose Response Using KLT

**Figure 7:**
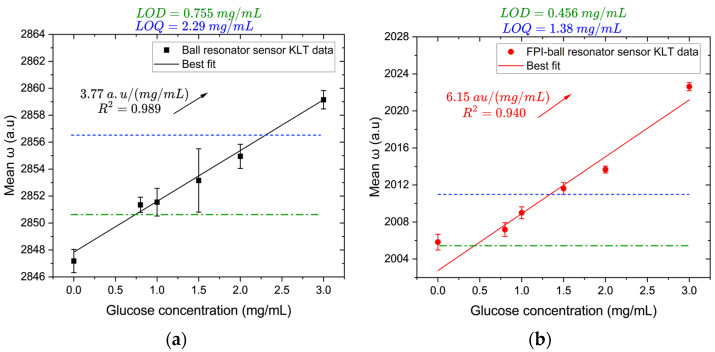
Glucose sensing performance (0–3 mg/mL) using KLT-demodulation: calibration curves for (**a**) the ball resonator sensor; and (**b**) the hybrid FPI–ball resonator sensor. Green and blue dashed lines indicate the LOD and LOQ values, respectively.

The spectral shifts in both sensors resulting from changes in glucose concentration are provided in Figure S2. As shown in Figure 7, both sensors exhibit a positive correlation between glucose concentration and the KLT-derived mean *ω* value. The ball resonator sensor (Figure 7a) exhibits a sensitivity of 3.77 ± 0.20 a.u./(mg/mL) with an excellent linearity (R^2^ = 0.989), a limit of detection (LOD) of 0.755 mg/mL, and a limit of quantification (LOQ) of 2.29 mg/mL. The slope is statistically significant (*p* < 0.001, 95% CI [3.21, 4.33]).

In comparison, the hybrid FPI–ball resonator sensor (Figure 7b) achieves a higher sensitivity of 6.15 ± 0.78 a.u./(mg/mL) while maintaining strong linearity (R^2^ = 0.940). The slope is statistically significant (*p* = 0.001, 95% CI [3.99, 8.31]). It also exhibits lower LOD and LOQ values of 0.456 mg/mL and 1.38 mg/mL, respectively. According to [43], most label-free fiber-optic glucose sensors show detection limits in the 3–10 mM range. The ball resonator sensor falls within this range with an LOD of 4.19 mM, whereas the hybrid FPI–ball resonator sensor surpasses typical performance with an LOD of 2.53 mM.

The enhanced sensitivity of the hybrid sensor arises from the presence of the FPI cavity, which converts bulk RI variations in the surrounding medium into significant interferometric phase shifts through changes in the optical path length of the cavity [44]. In the ball resonator sensor, changes in bulk RI influence the WGM resonance through its evanescent field, resulting in weaker sensitivity [6,10]. To confirm that the response to glucose arises from RI modulation, the relationship between mean *ω* values and RI of the glucose solutions (obtained from ORL 94BS refractometer) is provided in Figure S3. Both sensors exhibit a linear increase.

### 3.4. Thermal Response Using KLT

**Figure 8:**
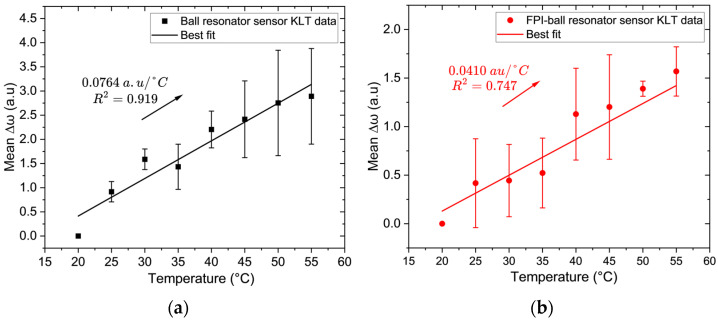
Temperature sensing performance (20–55 °C) obtained using KLT demodulation: (**a**) ball resonator sensor; and (**b**) hybrid FPI–ball resonator sensor.

Thermal response data were collected in 2 °C increments and averaged into 5 °C bins for clarity of presentation, as is commonly practiced in sensor characterization. Figure 8a shows the response of the ball resonator sensor to temperature variation. The sensor demonstrates a strong and reliable thermal response. The experimental *ω* values align very closely with the fitted line, yielding a sensitivity of 0.076 ± 0.006 a.u./°C with high linearity (R^2^ = 0.919), and also a statistically significant slope (*p* < 0.001, 95% CI [0.064, 0.089]). This indicates that KLT effectively extracts a stable temperature-affected feature from the spectrum of the ball resonator sensor.

The thermal response of the multi-cavity hybrid FPI–ball resonator sensor is shown in Figure 8b. The sensor exhibits a lower sensitivity of 0.041 ± 0.006 a.u./°C and reduced linearity (R^2^ = 0.747). The slope is statistically significant (*p* < 0.001, 95% CI [0.028, 0.054]). This behavior can be attributed to the hybrid structure, which contains multiple optical cavities whose responses to temperature differ due to thermo-optic and thermal expansion effects. The resulting complex spectral response is illustrated in Figure S4b. KLT minimizes this limitation by decomposing the spectral data into orthogonal components that emphasize the most statistically significant variations. Despite the challenges posed by its random spectral signature, the ball resonator sensor provides superior temperature sensitivity and reliability compared to the hybrid sensor.

### 3.5. pH Response Using KLT

**Figure 9:**
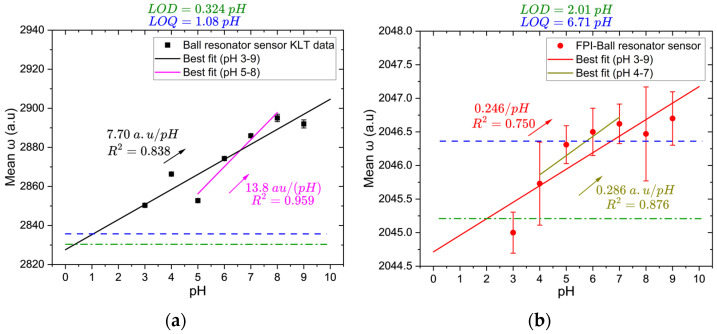
Sensing performance of sensors to pH using KLT demodulation: (**a**) the ball resonator sensor showing linear fits for the full range (pH 3–9) and the more linear sub-range (pH 5–8); (**b**) hybrid FPI–ball resonator sensor with linear fits for the full range (pH 3–9) and the more linear sub-range (pH 4–7). Green and blue dashed lines indicate the LOD and LOQ values, respectively.

KLT analysis successfully extracts a consistent pH-dependent response from the reflection spectra of both sensors. Figure 9a shows two segmented linear fits for the ball resonator sensor. Over the full range (pH 3–9), the sensor demonstrates a strong linear correlation between pH and the mean *ω* value, achieving a sensitivity of 7.70 ± 1.51 a.u./pH (*p* = 0.002, 95% CI [4.00, 11.40]) and R^2^ = 0.838. The calculated LOD and LOQ for this range are 0.324 and 1.08 pH units, respectively. Although these values lie outside the tested range (pH 3–9), they represent the theoretical detection limits extrapolated from the calibration curve. In the more linear sub-range (pH 5–8), the sensor exhibits an improved sensitivity of 13.8 ± 2.0 a.u./pH and R^2^ = 0.959, indicating that the ball resonator operates more predictably around neutral pH. This aligns with physiologically relevant conditions in biological tissues and wound healing environments. The sub-range is provided solely to illustrate the improved internal linearity; however, subsequent sensor performances are quantified from the full tested range (pH 3–9).

In contrast, as shown in Figure 9b, over the full range (pH 3–9), the hybrid FPI–ball resonator exhibits a considerably lower sensitivity of 0.25 ± 0.06 a.u./pH (*p* = 0.012, 95% CI [0.082, 0.410]) and reduced linearity (R^2^ = 0.750), with higher LOD and LOQ values of 2.01 and 6.71 pH units, respectively. A more linear sub-range (0.286 ± 0.076 a.u./pH, R^2^ = 0.876) is observed between pH 4–7, suggesting that the sensor is more reliable in mildly acidic to neutral conditions. The weaker performance of the hybrid configuration suggests that the FPI microcavity and the ball resonator respond differently to pH variations. Consequently, the net spectral shift becomes less predictable. Therefore, the ball resonator sensor provides more reliable detection of pH changes.

### 3.6. Multiparameter Sensing Capability

Overall, using KLT, the ball resonator sensor demonstrates superior performance for temperature and pH monitoring, whereas the hybrid FPI–ball resonator sensor exhibits higher sensitivity for glucose detection. This difference arises from the distinct sensing mechanisms governing each measurand and the spectral nature of the sensors. Glucose variation primarily induces changes in the bulk RI of the surrounding medium [11]. In the hybrid sensor, the FPI microcavity is highly sensitive to bulk RI changes. The microcavity converts bulk RI variations into significant optical path difference changes and phase modulations, resulting in enhanced sensitivity.

Temperature and pH variations, however, modify multiple optical parameters simultaneously. When tested in air, temperature changes affect the thermo-optic coefficient and thermal expansion of the silica fiber [45]. In the hybrid sensor, the FPI microcavity and the ball resonator respond differently to these thermal effects. As a result, their combined response leads to a less coherent spectral shift and reduced overall sensitivity. On the other hand, silanol groups on the silica surface exhibit amphoteric behavior and undergo protonation and deprotonation depending on pH. The resulting changes in surface charge density and subsequent ion adsorption modify the effective RI of the fiber [46]. The ball resonator sensor exhibits strong sensitivity to the change in effective RI due to its evanescent field interaction. In the hybrid sensor, the FPI microcavity introduces additional interference that reduces the pH-induced spectral response.

To address cross-sensitivity, a sensitivity matrix is established based on the sensitivity coefficients extracted from KLT analysis. The changes in *ω* values in the ball resonator $\left({\Delta \omega }_{ball}\right)$ and hybrid FPI–ball resonator $({\Delta \omega }_{hybrid})\;$sensors are expressed as linear combinations of the variations in the measurands. Although the sensors are physically distinct, they are subjected to the same external environment and therefore are treated as a single sensing system. Full decoupling of the three measurands is not achievable within this system with only two spectral outputs. However, reduced 2 × 2 sensitivity matrices can be constructed for one parameter pair at a time. For example, for changes in glucose (ΔG) and pH (ΔpH), the sensitivity matrix is written as:

(1) $$\left[\begin{array}{c} {\Delta \omega }_{ball}\\ {\Delta \omega }_{hybrid} \end{array}\right]\;=\left[\begin{array}{c} S_{ball,G}\;S_{ball,pH}\\ S_{hybrid,G}\;S_{hybrid,pH} \end{array}\right]\left[\begin{array}{c} \Delta G\\ \Delta pH \end{array}\right]$$

where $S_{ball,G}$ and $S_{ball,\;pH}$ represent the glucose and pH sensitivity coefficients of the ball resonator sensor, respectively, and $S_{hybrid,G}$ and $S_{hybrid,\;pH}$ denote the glucose and pH sensitivity coefficients of the hybrid FPI–ball resonator sensor, respectively. Substituting the sensitivity computation obtained from experimental results, Equation (1) can be rewritten as:

(2) $$\left[\begin{array}{c} {\Delta \omega }_{ball}\\ {\Delta \omega }_{hybrid} \end{array}\right]\;=\left[\begin{array}{cc} 3.8 & 7.7\\ 6.2 & 0.25 \end{array}\right]\left[\begin{array}{c} \Delta G\\ \Delta pH \end{array}\right]$$

The inverse of the matrix in Equation (2) enables decoupling of glucose and pH contributions. The condition number of the matrix is computed as the ratio of the largest to the smallest singular values obtained via SVD. A relatively large determinant (46.79) and a low condition number (1.86) indicate a well-conditioned system with stable inversion and robustness to measurement noise. These results demonstrate that even though each sensor exhibits cross-sensitivity to both measurands, their sensitivity coefficients are distinct enough to enable reliable discrimination between glucose and pH variations. In contrast, both sensors exhibit relatively weaker temperature sensitivity, which limits the reliable decoupling of temperature from glucose and pH responses.

In this work, signal demodulation is performed using only shifts in the dominant KLT-derived eigenvalue, which captures the primary spectral shifts. However, KLT provides multiple orthogonal eigenvalues, each capturing a distinct spectral feature. This provides a potential pathway to construct a higher-dimensional sensitivity matrix towards full multiparameter decoupling of the three measurands. Furthermore, the hybrid FPI–ball resonator sensor exhibits distinct spatial frequency components, whose response to multiple measurands can be effectively isolated using FFT-based filtering.

### 3.7. Comparison of KLT with Other Demodulation Methods

The results in Table 1 present the sensitivity of seven demodulation methods for glucose, temperature, and pH sensing. The conventional demodulation methods, centroid wavelength shift and intensity change, show inconsistent trends across sensors and measurands. For example, in glucose sensing, the centroid wavelength shift is positive for the ball resonator sensor but negative for the hybrid FPI–ball resonator sensor. This sign reversal arises because the ball resonator sensor is governed by Fano-like resonance, while the hybrid sensor exhibits periodic interference fringes. Consequently, each measurand perturbs the spectra of the sensors differently, leading to inconsistencies in the direction of the centroid shift and intensity change. Additionally, correlation-based methods (Pearson correlation and phase correlation) exhibit lower sensitivity because they implicitly assume rigid spectral shifts while preserving the shape. Practically, sensors undergo complex changes in spectral shape, contrast, and fringe visibility, which causes the correlation metrics to respond weakly and inconsistently to the underlying perturbations.

In contrast, the dimensionality reduction methods (KLT and PCA) and RMS provide more consistent trends across all measurands. These approaches are less affected by random spectral distortions because they operate on the full spectrum rather than relying on individual spectral features. Moreover, these methods transform the entire spectrum into a single scalar value. All three methods consistently deliver sensitivity responses with the same directional behavior for both sensors across all measurands. This indicates the robustness and improved reliability of these three methods in signal demodulation.

Consistent with the KLT analysis, the RMS analysis shows that the ball resonator sensor provides better temperature and pH monitoring capability, while the hybrid FPI–ball resonator sensor has better performance in monitoring glucose levels. In contrast, PCA shows that the ball resonator sensor outperforms the hybrid sensor for all three measurands. This observation highlights that the reported performance of the sensors can depend on the chosen demodulation method. However, a direct quantitative comparison among KLT, PCA, and RMS based on sensitivity is not meaningful because the output units and scales of these methods differ. Therefore, linearity (R^2^) was also used as a comparative metric.

**Figure 10:**
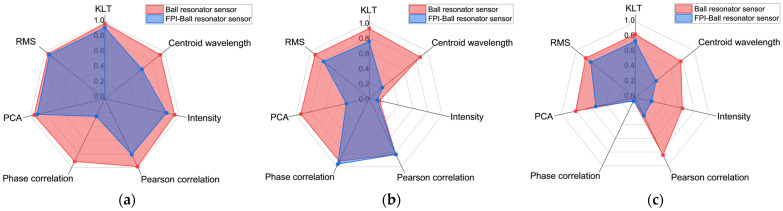
Radar plots comparing the performance based on linearity (R^2^) of the ball resonator and FPI–ball resonator sensor using seven spectral demodulation methods for monitoring: (**a**) glucose; (**b**) temperature; (**c**) pH.

The radar plots in Figure 10 provide a visual comparison of the performance of the seven demodulation methods in terms of R^2^. The red polygon, representing the ball resonator sensor, consistently encloses a larger area than the blue polygon representing the hybrid FPI–ball resonator sensor. This indicates that the ball resonator, with its single dominant cavity, shows stronger linearity and more predictable responses across all measurands. In glucose sensing, the hybrid FPI–ball resonator sensor exhibits competitive linearity using most demodulation methods, except centroid wavelength and phase correlation. For temperature and pH, the hybrid sensor’s R^2^ values are noticeably lower across most demodulation methods. This behavior suggests that the additional FPI cavity introduces complex interferences that reduce the sensor’s predictability. Furthermore, in both sensors, intensity change shows poor linearity for temperature sensing, while phase correlation performs poorly for pH detection. In contrast, KLT and RMS provide the highest linearity consistently across all three measurands in both sensors.

A summary is provided in Table 2 to compare the contribution of the current study with previous studies. Through a systematic benchmarking against six demodulation methods, KLT provides consistently superior robustness for demodulating both ball resonator and hybrid FPI–ball resonator OFSs, effectively overcoming the limitations associated with quasi-random and multi-cavity spectra. The robustness and automatic spectral feature extraction make KLT a powerful tool in optical fiber-based sensing.

A time-domain drift analysis was performed by monitoring changes in *ω* of both sensors over a 4-h period under a controlled thermal condition and immersing both sensors in a 1 mg/mL glucose solution. The ball resonator sensor exhibited a drift rate of approximately 0.215 a.u./h, while the hybrid FPI–ball resonator sensor showed a slightly higher drift of −0.382 a.u./h (Figure S6). The drift rates were normalized with respect to the initial *ω* values and expressed as relative drift rates (%/h). The relative drift rates were estimated to be approximately 0.0076%/h for the ball resonator and 0.0184%/h for the hybrid sensor. Both relative drift rates are below 0.02%/h, demonstrating low signal drift and good short-term stability of the sensors.

## 4. Conclusions

When compared to other types of OFSs, ball resonator sensors offer simplicity, rapid fabrication, and mechanical robustness. However, their low-contrast and quasi-random reflection spectra hinder their biosensing applications. Our findings underscore that KLT provides a significant performance enhancement for signal demodulation in both a ball resonator and a hybrid FPI–ball resonator sensor. KLT-based demodulation outperformed the conventional and correlation-based approaches in glucose, temperature, and pH sensing. These results confirm that KLT is a robust and computationally efficient demodulation approach for multiparameter sensing. The hybrid sensor shows enhanced glucose sensitivity, achieving a 2.53 mM LOD. The ball resonator offers superior thermal and pH sensitivity with R^2^ of 0.919 and 0.838, respectively.

## Figures and Tables

**Table 1:** Sensitivity of the ball resonator and FPI–ball resonator sensor for glucose, temperature, and pH using seven spectral demodulation methods.

Analysis Method	Glucose (/(mg/mL))		Temperature (/°C)		pH (/pH)	
	Ball Resonator	FPI–Ball Resonator	Ball Resonator	FPI–Ball Resonator	Ball Resonator	FPI–Ball Resonator
Centroid wavelength (pm)	190 ± 30	−42 ± 16	3.9 ± 0.4	−0.97 ± 0.45	255 ± 63	−5.6 ± 3.4
Intensity (dB)	−0.029 ± 0.004	−0.057 ± 0.013	(−6.8 ± 0.4) × 10^−4^	(1.3 ± 0.9) × 10^−3^	−0.11 ± 0.04	(5.6 ± 4.7) × 10^−^^3^
Pearson correlation (a.u)	−0.014 ± 0.001	(−1.5 ± 0.4) × 10^−4^	(1.3 ± 0.2) × 10^−4^	(−6.8 ± 0.8) × 10^−6^	−0.031 ± 0.006	(−4.1 ± 3.0) × 10^−6^
Phase correlation (pm)	1.2 ± 0.2	0.37 ± 0.32	0.045 ± 0.003	0.18 ± 0.01	7.6 ± 5.6	−0.033 ± 0.064
PCA (a.u)	−8.5 ± 1.0	−7.1 ± 1.1	−0.20 ± 0.01	−0.022 ± 0.008	−11.4 ± 2.48	−0.34 ± 0.14
RMS (dB)	0.033 ± 0.004	0.064 ± 0.009	(9.1 ± 0.7) × 10^−4^	(4.9 ± 0.6) × 10^−4^	0.072 ± 0.014	(2.7 ± 0.7) × 10^−^^3^
KLT (a.u)	3.8 ± 0.2	6.2 ± 0.8	0.076 ± 0.006	0.041 ± 0.006	7.7 ± 1.5	0.25 ± 0.06

**Table 2:** Review of relevant previous studies on OFSs and the present work in terms of sensor architecture, spectral type, presented demodulation approaches, and multiparameter sensing capability.

Reference	Sensor Architecture	Spectral Type	Demodulation Method	Multiparameter Measurement
Tosi et al. 2021 [5]	ball resonator	non-periodic	2 (feature extraction and KLT)	No (RI/biomarker)
Shaimerdenova et al. 2020 [13]	ball resonator	non-periodic	2 (wavelength and amplitude tracking)	No (RI)
Tosi 2015 [26]	FBG, FPI, and hybrid FBG-FPI	narrowband and periodic	3 (bandwidth tracking, Q-point tracking, and KLT)	No
Liu et al. 2022 [47]	FPI	periodic	2 (KLT and SVD)	No (pressure)
Zhong et al. 2022 [48]	fiber grating	multiple spectral dips	1 (PCA)	Yes (strain and torsion)
This work	ball resonator and hybrid FPI–ball resonator	non-periodic and periodic	7 (centroid wavelength shift, intensity change, Pearson correlation, phase correlation, PCA, RMS, and KLT)	Yes (glucose, pH, temperature)

## Data Availability

The original contributions presented in this study are included in the article/Supplementary Material. Further inquiries can be directed to the corresponding author.

## Supplementary Materials

The following supporting information can be downloaded at: https://www.mdpi.com/article/10.3390/bios16050278/s1, Table S1: Fusion splicer parameters used for ball resonator fabrication using the Fujikura FSM-100P+ fusion splicer. Figure S1: Variation in RI of the glucose solutions as a function of glucose concentration (mg/mL). Figure S2: Spectral shift in the sensors with increasing glucose concentration: (a) ball resonator sensor; (b) hybrid FPI–ball resonator sensor. Figure S3: Sensitivity of sensors to RI variation using KLT demodulation: (a) ball resonator sensor; (b) hybrid FPI–ball resonator sensor. Figure S4: Spectral shift in the sensors with changes in temperature: (a) ball resonator sensor; (b) hybrid FPI–ball resonator sensor. Figure S5: Spectral shift in the sensors due to changes in pH: (a) ball resonator sensor; (b) hybrid FPI–ball resonator sensor. Figure S6: KLT eigenvalue (*ω*) drift plots over 4 h under controlled conditions: (a) ball resonator sensor; (b) hybrid FPI–ball resonator sensor.
