# Supplementary material for: Systematic Benchmarking of Spectral Demodulation Methods for Ball Resonator and Hybrid FPI–Ball Resonator Sensors for Multiparameter Physiological Monitoring
Source: Biosensors (Basel). 2026 May 11;16(5):278. doi: 10.3390/bios16050278 (PMC13204527; doi:10.3390/bios16050278)
Supplement: Supplementary file 1 [file biosensors-16-00278-s001.zip › biosensors-4227352-supplementary.pdf]

biosensors

MDPI

Article

Systematic Benchmarking of Spectral Demodulation Methods for Ball Resonator and Hybrid FPI–Ball Resonator Sensors for Multiparameter Physiological Monitoring

Natsnet Bereket Tecle [1], M. Fátima Domingues [1,2,3*]

[1] Department of Biomedical Engineering and Biotechnology, Khalifa University of Science & Technology, Abu Dhabi 127788, United Arab Emirates
[2] Healthcare Engineering and Innovation Group, Khalifa University of Science & Technology, Abu Dhabi 127788, United Arab Emirates
[3] Department of Electrical and Computer Engineering, Instituto de Telecomunicações, University of Aveiro, Aveiro 3810-193, Portugal
* Correspondence: fatima.domingues@ua.pt

Table S1. Fusion splicer parameters used for ball resonator fabrication using the Fujikura FSM-100P+ fusion splicer.

Parameter	Value
Cladding diameter	125 µm
Cleave length	11 mm
Electrode gap	2.2 mm
Gap distance	15 µm
Shaping preset	NS F125 B300
Arc power	STD[311] bit
Arc time	2000 ms
Rearc Power	STD +100 bit
Rearc Time	800 ms
Rearc On Time	800 ms
Alignment mode	Cladding
Operating method	PAS
Operating mode	Fiber shaping

Received: 13 March 2026
Revised: 1 May 2026
Accepted: 6 May 2026
Published: 11 May 2026

Copyright: © 2026 by the authors. Licensee MDPI, Basel, Switzerland. This article is an open access article distributed under the terms and conditions of the Creative Commons Attribution (CC BY) license.

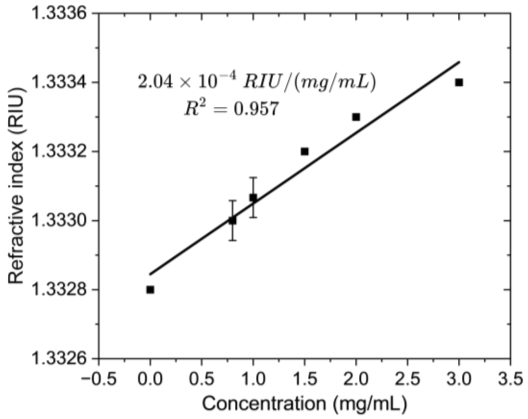

Figure S1. Variation of RI of the glucose solutions, measured using an ORL 94BS refractometer, as a function of glucose concentration (mg/mL).

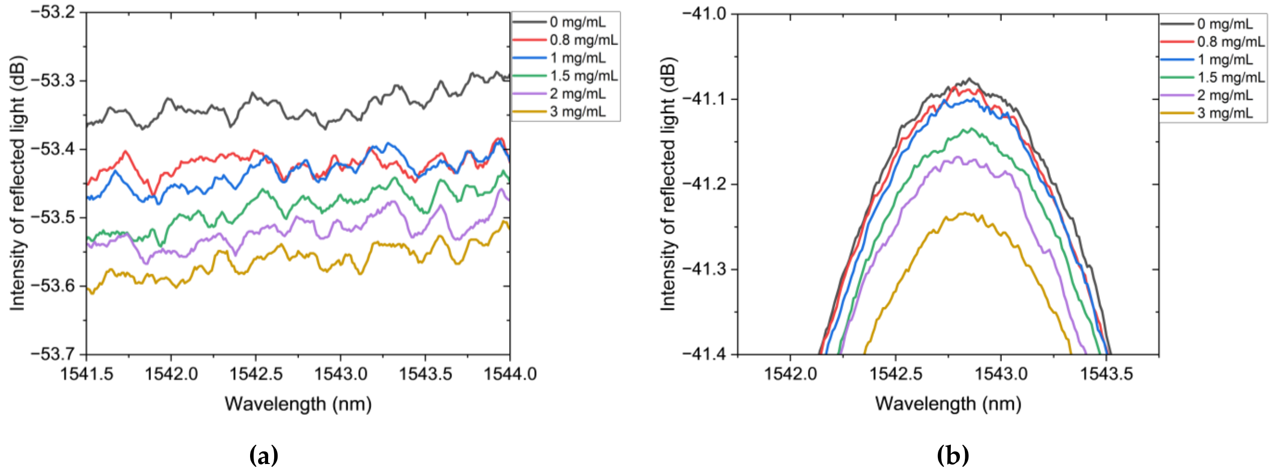

Figure S2. Spectral shift of the sensors with increasing glucose concentration: **(a)** ball resonator sensor; **(b)** hybrid FPI–ball resonator sensor.

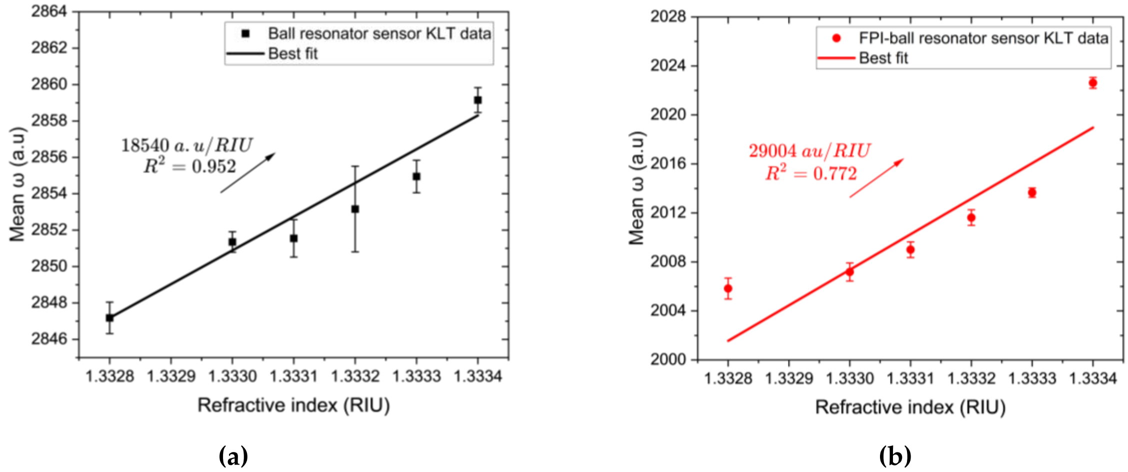

Figure S3. Sensitivity of sensors to RI variation (from 1.3328 to 1.3334 RIU) using KLT demodulation: **(a)** ball resonator sensor; **(b)** hybrid FPI–ball resonator sensor.

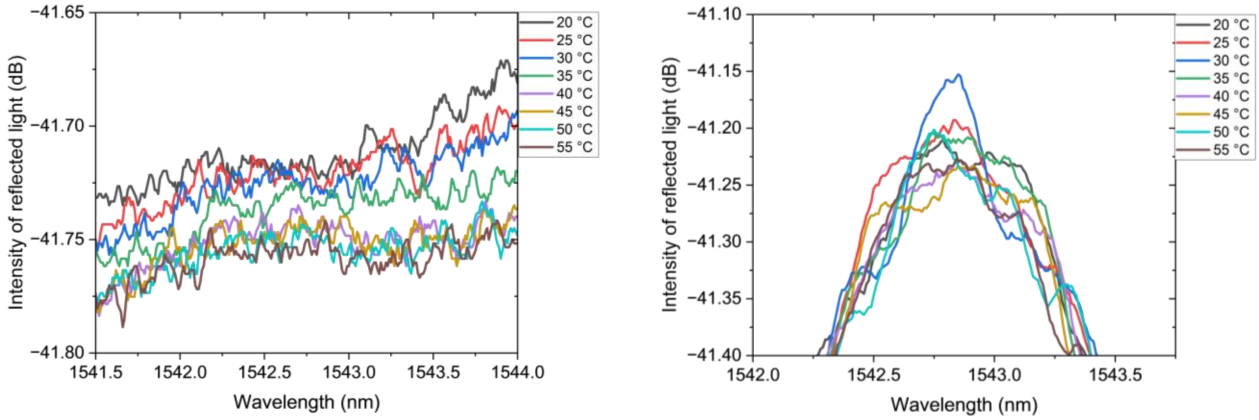

(a) **(b)**

Figure S4. Spectral shift of the sensors with changes in temperature: **(a)** ball resonator sensor; **(b)** hybrid FPI–ball resonator sensor.

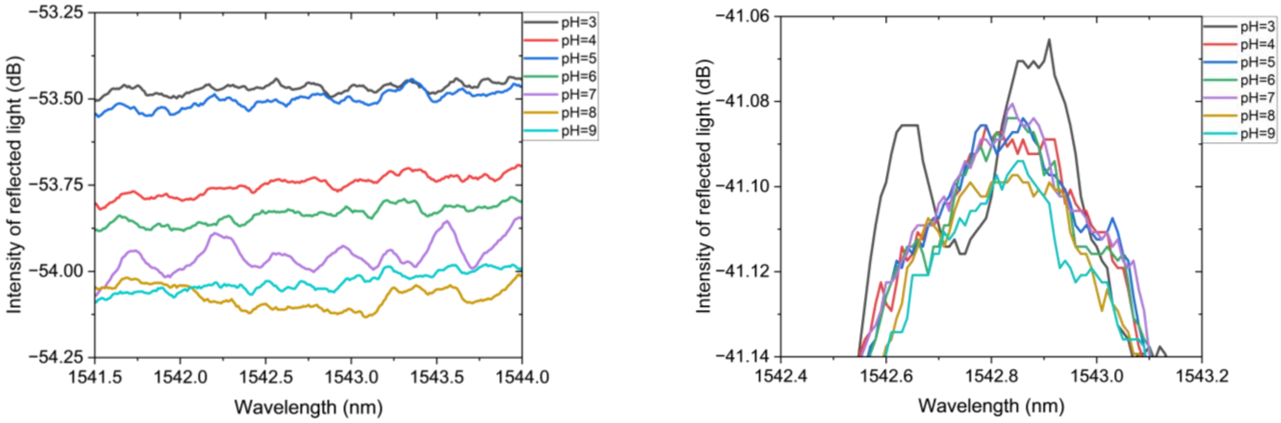

(a) **(b)**

Figure S5. Spectral shift of the sensors due to changes in pH: **(a)** ball resonator sensor; **(b)** hybrid FPI–ball resonator sensor.

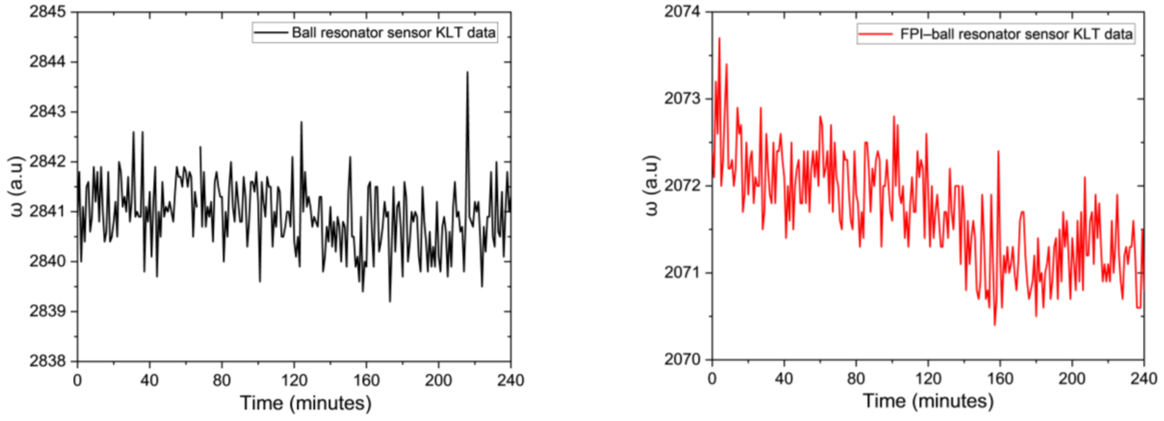

(a) **(b)**

Figure S6. KLT eigenvalue (ω) drift plots over 4 hours under controlled conditions: **(a)** ball resonator sensor; **(b)** hybrid FPI–ball resonator sensor.
